# A bicentric retrospective study of the correlation of EAU BCR risk groups with ^18^F-PSMA-1007 PET/CT detection in prostate cancer biochemical recurrence

**DOI:** 10.1038/s41598-024-61121-3

**Published:** 2024-05-13

**Authors:** Nathan Poterszman, Charles Merlin, Charles Margail, Eric Ouvrard, Alessio Imperiale, François Somme

**Affiliations:** 1 Nuclear Medicine and Molecular Imaging, Institut de Cancérologie Strasbourg Europe, 17 Rueueue Albert Calmette, 67000 Strasbourg, France; 2 Nuclear Medicine, Centre Jean Perrin, Clermont-Ferrand, France; 3 Molecular Imaging-DRHIM, IPHC, UMR 7178, CNRS/Unistra, Strasbourg, France

**Keywords:** Prostate cancer, Diagnostic markers, Molecular medicine

## Abstract

The European Association of Urology (EAU) has proposed a risk stratification for patients harboring biochemical recurrence (BCR) after radical prostatectomy: ISUP < 4 and PSA doubling time (PSAdt) > 12 months for low risk, and ISUP ≥ 4 or PSAdt ≤ 12 months for high risk. This dual-center retrospective study aims to investigate the correlation between the EAU risk stratification for BCR following radical prostatectomy and the detection rate of lesions using ^18^F-PSMA-1007 PET/CT. Among the 71 included patients (58 high-risk, 13 low-risk), with a median PSA level of 1.43 ng/ml, PET/CT demonstrated a significantly higher positivity in the high-risk group compared to the low-risk group (72.4% vs. 38.0%, *p* = 0.026). Analysis of recurrence sites revealed a similar proportion of pelvic-confined disease in both groups (24.1% vs. 23.1%, p = 0.935), but a significantly higher incidence of metastatic disease in the high-risk group (51.7% vs. 15.4%, p = 0.017), with detailed findings indicating an increased prevalence of bone metastases in the high-risk BCR group (37.8% vs. 7.7%, p = 0.048). Therefore, PSMA PET/CT offers valuable insights for treatment decisions, aligning with the evolving landscape of prostate cancer management.

## Introduction

The diagnostic performance of radiolabeled PSMA PET/CT in patients with a biochemical recurrence (BCR) of prostate cancer (serum PSA greater than 0.2 ng/ml after prostatectomy or nadir PSA greater than 2 ng/ml after radiotherapy^1^) is well-established. The detection rate ranges from 47 to 95%^2^ justifying the recent inclusion of radiolabeled PSMA PET/CT in the 2022 EAU-ESTRO-EANM-ESUR-SIOG prostate cancer guidelines^3^. However, although the tumor detection rate of PSMA PET/CT appears to correlate with both PSA value and PSA doubling time g(PSA-DT)^2^, there is currently no clear consensus on when to perform PSMA PET/CT in patients with a biochemical recurrence.

The goal of the radical prostatectomy is the eradication of cancer while, whenever possible, preserving pelvic organ function. The procedure involves removing the entire prostate with its capsule intact and seminal vesicles, followed by vesicourethral anastomosis. Surgical approaches have expanded from perineal and retropubic open approaches to laparoscopic and robotic-assisted techniques. The European Association of Urology (EAU) proposed a two-risk group BCR stratification based on the following criteria after radical prostatectomy: PSA-DT > 1 year and International Society of Urological Pathology (ISUP) grade < 4 for low-risk patients, and PSA-DT ≤ 1 year or ISUP 4–5 for high-risk^4^. Neither the EAU criteria nor the meta-analysis from Van den Broeck et al*.* took into account PSMA PET/CT. The correlation between the EAU criteria and PSMA PET/CT was carried out on 94 patients imaged by ^18^F-DCFPyL and 51 by ^68^Ga-PSMA-11^5^ and furthermore evaluated in 1960 patients in an international multicenter study with ^68^Ga-PSMA-11^6^. It demonstrated that patients within the EAU high-risk group were more likely to have a PSMA PET/CT metastatic disease (37% vs 24%). The discovery of a metastatic disease led to a significant management change for physicians.

Gallium-based radiotracers require an on-site generator and the amount of elution per day is limited. As prostate cancer is the most common cancer in men, nuclear medicine centers need to be able to meet the growing demand for PSMA PET/CT. On the opposite, ^18^F-PSMA-1007 presents several advantages making it an interesting alternative. It is easier to supply and its low urinary excretion allows for a quality analysis of the pelvis. Another study has already evaluated 194 patients in the setting of primary staging of prostate cancer^7^. They demonstrated that a metastatic disease on PSMA PET/CT was associated with ISUP grade and PSA level. In the setting of the biochemical recurrence, there is a lack of data on the correlation between the ^18^F-PSMA-1007 PET/CT detection rate and PSA level or ISUP grade.

The purpose of this study was to analyze the correlation between the EAU BCR risk group and the detection rate of an ^18^F-PSMA-1007 PET/CT.

## Methods

### Patients

This is a dual-center non-interventional retrospective study involving patients with a BCR of prostate cancer after a radical prostatectomy with curative intent defined as a serum PSA greater than 0.2 ng/ml. The included patients underwent an ^18^F-PSMA-1007 PET/CT in the Nuclear Medicine Departments of the Institut de Cancerologie Strasbourg (ICANS), Strasbourg, and the Centre Jean Perrin (CJP), Clermont-Ferrand. Only patients with PSA values and available histology who could thus be classified according to the EAU BCR risk groups were included. PSA doubling time was calculated using the last two values before PSMA PET/CT (with the Memorial Sloan Kettering Cancer Center tool^8^). ISUP grade was retrieved from the histological analyses.

The institutional review board (Commission d’Orientation Recherche et Enseignement of the Institut de Cancerologie de Strasbourg) has approved the study. Informed consent was obtained from all participants. All research was performed in accordance with relevant guidelines and in accordance with the Declaration of Helsinki.

### Imaging protocols

^18^F-PSMA-1007 PET/CT was performed using a combined PET/CT device (Biograph Vision 600, Siemens and Discovery MI, General Electric). A 2 MBq/kg dose of ^18^F-PSMA-1007 was intravenously injected. Whole-body (top of the skull to the upper thigh) PET/CT was performed 60 min after injection. Neither contrast agents nor diuretics (furosemide) were used. PET data were reconstructed iteratively (OSEM algorithm) using CT data for attenuation correction. CT, PET (after attenuation correction), and PET/CT fusion images were displayed on a dedicated workstation for analysis (Syngo.via VB30B, Siemens).

### Image analysis

Each ^18^F-PSMA PET/CT has been reinterpreted using the E-PSMA v1.0 guidelines^9^. In brief, the 5-point scale was used to rate each examination. E-PSMA 1 and 2 were classified as negative ^18^F-PSMA PET/CT whereas E-PSMA 4 and 5 were classified as positive. Considering E-PSMA 3, two experienced nuclear physicians reviewed the images to reach a consensus for negative or positive classification. This method was used to reduce the number of false-positive studies, especially due to unspecific bone uptake that can be present in up to 50% of patients^10^. The pelvis-confined disease was defined as a prostate bed recurrence or pelvic lymph node beneath the common iliac areas. Metastatic disease was defined as the involvement of the common iliac lymph nodes or above the iliac bifurcation, bone, or visceral PSMA-positive lesions. This relied on the PROMISE classification^11^.

### Statistical analysis

The Statistical analyses were made with Jamovi (version 2.4.11; computer software). χ^2^ test was conducted to compare groups of patients. It was replaced by Fisher’s exact test when conditions of validity were not met. A significant level of 5% was used. Logistic regression models were used for univariate and multivariate analyses (adjusted for potential confounding factors). Risk ratios were calculated with a 95% confidence interval.

### Ethics approval and consent to participate

The need for ethics approval and consent to participate was waived.

## Results

From June 2021 to February 2023, 108 patients underwent an ^18^F-PSMA-1007 PET/CT. Seventy-one fit our inclusion criteria (60 at ICANS and 11 at CJP). Fifty-eight of them presented a high-risk BCR and 13 a low-risk BCR. Patients in the two groups were not significantly different in terms of age, adjuvant radiation therapy, PSA at the time of the PET/CT (with a median PSA level of 1.43 ng/ml; interquartile 0.736–2.77), the time interval between radical prostatectomy and the PSMA PET/CT (with a median time of 7.2 years; interquartile 3.7–13.1), and the initial stage of the disease (TNM). The characteristics of both groups are detailed in Table 1.

**Table 1:** Characteristics of patients in low and high-risk BCR groups.

	Low-risk BCR group (n, %)	High-risk BCR group (n, %)	*p*-value
Number of patients	13 (18.3)	58 (81.7)	
Age (median, y)	70.2	70	0.947
Cohort			0.108
ICANS	9 (12.7)	51 (71.8)	
Centre Jean Perrin	4 (5.6)	7 (9.9)	
PSA (median, ng/ml)	1.32	1.49	0.339
< 0.5 (%)	23.1	15.5	
0.5—1 (%)	23.1	19	
> 1 (%)	53.8	65.5	
**PSA doubling time (month)**			** < 0.01**
< 12 (%)	0 (0)	56 (96.4)	
≥ 12 (%)	13 (100)	2 (3.6)	
**ISUP score**			**0.029**
< 4 (%)	13 (100)	41 (70.9)	
≥ 4 (%)	0 (0)	17 (29.1)	
pT stage			0.757
< pT3 (%)	5 (38.5)	28 (48.1)	
≥ pT3 (%)	8 (61.5)	30 (51.9)	
Adjuvant radiotherapy	11 (84.6)	45 (77.6)	0.721

*BCR* bioChemical recurrence.

Significants values are in bold.

At least one PSMA-positive lesion was visualized in 47 (66.2%) patients. Nine (12.7%) examinations were classified as E-PSMA 1 or 2 and 39 (54.9%) as E-PSMA 4 or 5. A key point was the significant number of E-PSMA 3 PET/CT, i.e. 23 (32.4%). Fifteen (65.2%) were finally rated negative. All examinations rated negative were due to an unspecific bone uptake, with two patients also having false-positive pelvic lymph node uptake (due to inguinal lymph nodes ultimately rated as negative).

**Figure 1:**
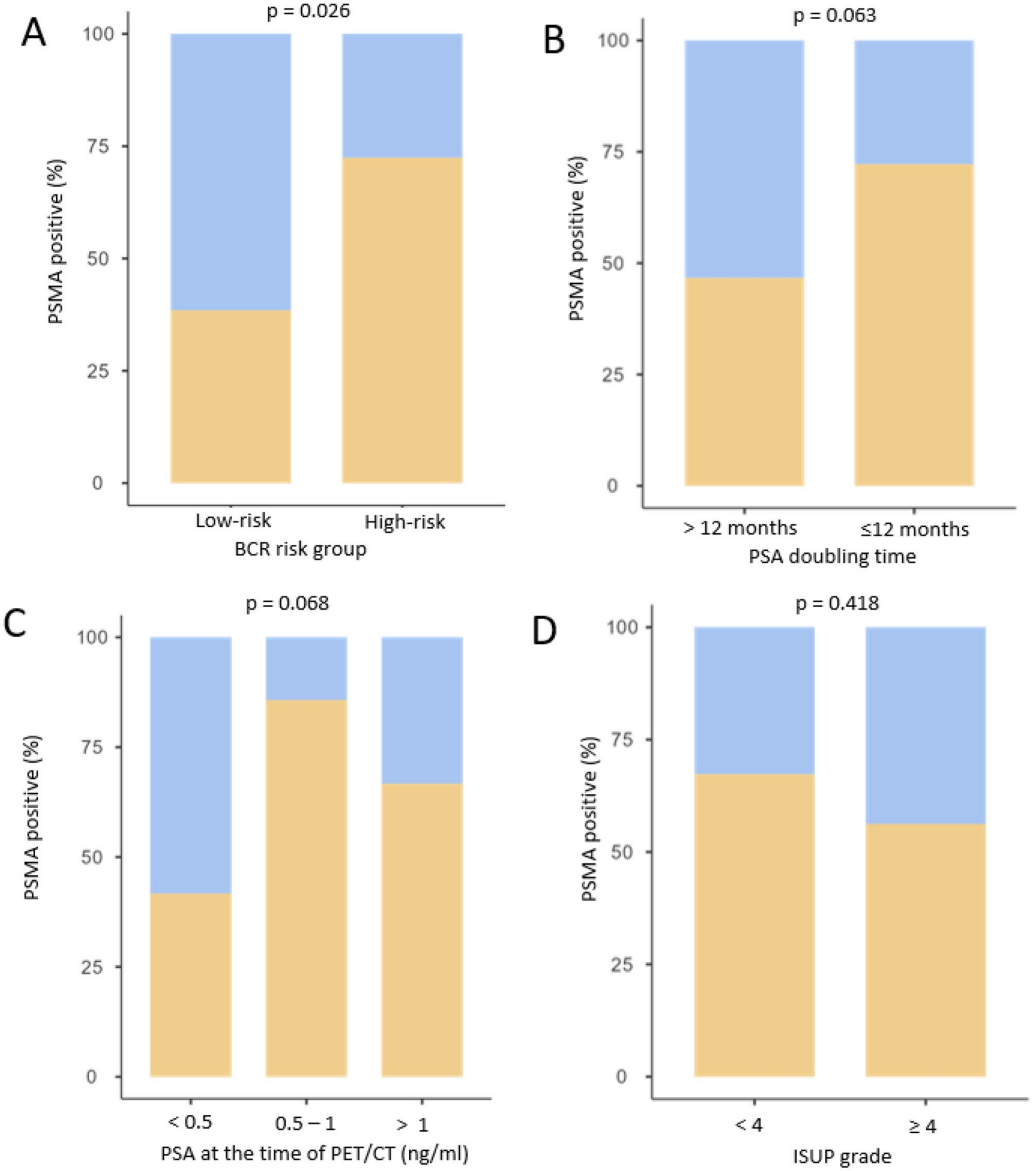
Percentage of patients with at least one (yellow) or no (blue) PSMA-positive lesion on PET/CT according to their BCR risk-group (**A**), PSA doubling time (**B**), PSA value at the time of the examination (**C**) or ISUP grade (**D**).

PSMA PET/CT positivity was significantly higher in the EAU high-risk BCR versus the low-risk group (72.4% vs 38.0%, *p* = 0.026, Fig. 1A)*.* The risk ratio between the two groups was 2.23 (95% CI, 1.23–4.06). In univariate analysis, no correlation was found between the positivity of the PSMA PET/CT and the PSA doubling time (*p* = 0.063; Fig. 1B) neither the PSA value at the time of PET/CT ((*p* = 0.068; Fig. 1C). No significant correlation was observed between the positivity of the PSMA PET/CT and the ISUP grade (Fig. 1D), nor adjuvant radiation therapy.

The multivariate logistic regression analysis (adjusted for age, center, PSA at the time of PET/CT, PSA doubling time, TNM stage, and ISUP grade) confirmed that the EAU BCR risk group was the sole predictive factor for a positive ^18^F-PSMA-1007 PET/CT (*p* = 0.013) in our cohort.

The patient’s outcome and therapeutic management are heavily influenced by the presence of metastatic dissemination. Thus, we further analyzed the localization of the recurrence. Seventeen patients (23.9%) out of the 71 had a pelvis-confined (prostate bed or pelvic lymph node) disease with a similar proportion in both BCR risk groups (25.9% vs 23.1%, *p* = 1.0). Thirty-two patients (45.1%) had metastatic disease with a significantly higher rate in the high-risk BCR group (51.7 % vs 15.4%, *p* = 0.017). In detail, considering the recurrence site, a similar proportion of patients presented a prostate bed recurrence in both groups (15.4% vs 19.0% in low and high-risk respectively, *p* = 1.0). It is interesting to note that out of 12 patients who experienced a prostate bed recurrence, 11 (91.7%) had not undergone salvage radiotherapy. No significant difference was found in terms of lymph node involvement (23.1% vs 41.4*%, p* = 0.219). Interestingly, a higher proportion of patients had bone metastasis in the high-risk BCR group (7.7% vs 37.8%, *p* = 0.048). Visceral metastases were too rare to draw any conclusions. Two lung metastases were found in the high-risk BCR group; no visceral metastasis was observed in the low-risk BCR group. Main results are summarized in Table 2.

**Table 2:** Results of PSMA PET/CT according to low and high-risk BCR groups.

	Low-risk BCR group (%)	High-risk BCR group (%)	*p*-value
**PSMA PET/CT positivity**	38.0	72.4	**0.026**
Pelvis-confined disease	23.1	24.1	0.935
**Metastatic disease**	15.4	51.7	**0.017**
Prostate bed recurrence	15.4	19.0	1.0
Lymph node metastasis	23.1	41.4	0.219
**Bone metastasis**	7.7	37.8	0**.048**

BCR bioChemical recurrence.

Significants values are in bold.

## Discussion

In this retrospective bicentric study, we underscored the significance of ^18^F-PSMA-1007 PET/CT in patients experiencing biochemical recurrence (BCR) after radical prostatectomy, particularly within the subgroup classified as high-risk according to the EAU criteria. Notably, individuals with a high-risk BCR demonstrated a higher incidence of ^18^F-PSMA-positive lesions compared to those with a low-risk BCR.

Our findings align with similar results reported in two other studies that utilized different PSMA ligands, namely ^18^F-DCFPyL and ^68^Ga-PSMA. Dong et al. observed a significantly higher positive rate in high-risk BCR patients in comparison to low-risk cases (82.0% versus 48.9%, *p* < 0.001)^5^. Additionally, Ferdinandus et al. documented a higher prevalence of M1 disease in the high-risk group compared to the low-risk group (37% versus 24%, *p* < 0.001)^6^. 

The ARTISTIC meta-analysis demonstrated that the systematic use of adjuvant radiation therapy following prostatectomy does not improve PSA-driven event-free survival in men with localized or locally advanced prostate cancer^12^. Therefore, early salvage radiation therapy (SRT) is now the recommended treatment in this situation. The fundamental idea behind the EAU BCR risk profile revolves around questioning whether adjuvant SRT should be pursued or not. Indeed, Preisser et al*. *analyzed 2379 patients (805 low-risk and 1574 high-risk BCR) with a median follow-up of 54 months^13^. For low-risk BCR, 12-year overall survival was 87% versus 78% (*p* = 0.2) and cancer-specific survival was 100% versus 96% (*p* = 0.2) for early versus no SRT. For high-risk BCR, 12 year overall survival was 81% versus 66% (*p* < 0.001) and cancer-specific survival was 98% versus 82% (p < 0.001) for early versus no SRT. The authors concluded that while men with high-risk BCR should be offered SRT, surveillance might be a suitable option for those with low-risk BCR. Thus, PSMA PET/CT represents an additional tool to provide evidence in favor of surveillance in patients with low-risk BCR and a negative examination. 

A noteworthy strength of our study lies in the consistent use of a single fluor-based radiotracer (^18^F-PSMA-1007) across the entire cohort. Unlike gallium-based radiotracers, which necessitate an on-site generator and have limited daily elution capacity, the use of ^18^F-PSMA-1007 provides practical advantages for broader clinical application. Despite similarities in behavior among PSMA-based PET radiotracers, certain distinctions persist. Notably, ^18^F-PSMA-1007 exhibits significant biliary excretion, while ^68^Ga-PSMA-11 is predominantly excreted in the urinary tract^14^. These differences justified the exploration of the value of a fluor-based radiotracer in this specific clinical context.

Regarding the site-based analysis in our study, there are two significant points. Firstly, the management of pelvic nodal recurrence alone is still controversial. Falkenbach et al*. *classified 222 patients into the EAU BCR risk group to assess their influence on the outcomes of radioguided surgery against prostate-specific membrane antigen^15^, among which 57.7% of patients had previously undergone salvage radiotherapy. Authors concluded that neither EAU criteria nor the kinetic parameters of PSA were correlated with BCR-free and therapy-free survivals. However, in this study, 39 patients (17.4%) presented with retroperitoneal lymph nodes, thus classifying them in the metastatic category and potentially explaining the study’s results. In our cohort, 9 patients (19.1%) harbored a pelvic nodal recurrence alone. These are the patients who could benefit the most from a metastasis-directed therapy after (such as radioguided surgery or stereotactic radiotherapy). We still lack data on this issue as most studies were conducted using conventional imaging or choline PET/CT. Secondly, it is noteworthy that patients in the high-risk BCR group exhibited a significantly higher prevalence of PSMA-positive bone lesions in our study. Although ^18^F-PSMA is known for a higher rate of false-positive bone uptake compared to other PSMA radioligands^16^, the systematic use of the E-PSMA 5-point scale in our study likely contributed to reducing the rate of false-positive examinations.

Contrary to expectations based on other studies, our investigation did not reveal a significant difference in lymph node involvement between high-risk and low-risk groups. This discrepancy could be attributed to a unique aspect of the PSMA PET/CT process in France. Specifically, patients in our study had to undergo a negative ^18^F-choline PET/CT before proceeding to a PSMA PET/CT. This selection criterion distinguishes our study from its international counterparts.

It is intriguing to note that despite the EAU risk group criteria relying on biological and clinical data, our study demonstrated consistency in predicting PET PSMA findings. In contrast to our results, factors such as PSA doubling time (PSA-DT) and ISUP grade, known for their prognostic significance^17,18^, exhibited a correlation with metastatic disease detected on PSMA PET/CT in the literature. This suggests a logical correlation between two prognostic indicators—EAU risk groups and the presence of metastatic disease on PSMA PET/CT.

While a high PSA value has previously been identified as a robust predictor of a positive PSMA PET/CT result^2^, our study did not establish a significant correlation between PSA value and PET/CT positivity in our cohort. The limited sample size likely contributed to this inconclusive result, restricting the feasibility of subgroup analyses.

## Conclusion

Our study underscored a higher incidence of ^18^F-PSMA-positive lesions in EAU high-risk BCR patients. Notably, PSMA PET/CT offers valuable insights for treatment decisions, aligning with the evolving landscape of prostate cancer management. The clinical implications of these findings remain to be explored.

## Data Availability

The datasets used during the current study are available from the corresponding author on reasonable request.
